# Your CT scans are hiding crucial TAVI survival data: Are you looking?

**DOI:** 10.1016/j.ahjo.2025.100649

**Published:** 2025-10-21

**Authors:** Marek Kantor, Otakar Jiravsky, Matej Pekar

**Affiliations:** a Complex Cardiovascular Center, Hospital AGEL Třinec-Podlesí, Konská 453, 739 61, Třinec, Czech Republic; b Medicine, Faculty of Medicine, University of Ostrava, Syllabova 19, 70300, Ostrava, Czech Republic; c Department of Physiology, Faculty of Medicine, Masaryk University, Kamenice 5, 625 00, Brno, Czech Republic

**Keywords:** Transcatheter aortic valve replacement, Computed tomography, Adipose tissue, Body composition, Risk stratification, Diagnostic imaging, Cardiology, Risk assessment, Geriatrics

## Abstract

Transcatheter Aortic Valve Implantation (TAVI) has revolutionized treatment for severe aortic stenosis, but optimal patient selection remains challenging. This commentary highlights findings from our recent systematic review of 14 studies comprising 9692 TAVI patients, which revealed that CT-derived adipose tissue parameters provide valuable prognostic information often overlooked during procedural planning. We found that higher subcutaneous adipose tissue consistently associated with better survival, while adipose tissue quality, measured by CT attenuation, proved equally important. The relationship between adiposity and outcomes appears U-shaped rather than linear, with both extremely low and high adiposity quartiles correlating with worse outcomes, while moderate subcutaneous adiposity provides optimal outcomes by offering metabolic reserves without pathological complications. Notably, fat distribution patterns (VAT:SAT ratio < 1) were associated with better cardiovascular outcomes, underscoring that where fat is stored matters more than total quantity. The obesity-dependent effects of visceral adipose tissue reflect fundamental differences in metabolic physiology: in non-obese patients, modest VAT represents protective energy reserves, while in obese patients, lower VAT indicates relatively better metabolic health within the context of existing obesity. These adipose tissue characteristics are readily available in pre-procedural CT scans already used for anatomical assessment, requiring minimal additional resources while potentially enhancing risk stratification. We present a novel decision algorithm with sex-specific thresholds that enables immediate clinical implementation of these measurements for patient risk stratification. As TAVI indications expand to include both increasingly frail elderly patients and those at intermediate surgical risk, integrating these overlooked adipose tissue parameters into clinical decision-making could improve patient selection and outcomes.

Transcatheter Aortic Valve Implantation (TAVI) has revolutionized treatment for severe aortic stenosis, expanding therapeutic options for high-risk patients. As indications broaden to include both increasingly frail elderly patients and those at intermediate surgical risk, precise risk stratification becomes increasingly critical for structural heart teams. Our recent systematic review examining CT-derived adipose tissue parameters in 9692 TAVI patients revealed that clinicians may be overlooking valuable prognostic information hidden in the very CT scans already used for procedural planning [1].

While our recently published systematic review established the evidence base for CT-derived adipose tissue parameters in TAVI outcomes, this commentary serves a fundamentally different purpose: translating research findings into immediately actionable clinical practice. Critically, this commentary focuses on extracting valuable prognostic information from imaging studies already performed as standard care, requiring no additional testing burden, radiation exposure, or procedural modifications.

Structural heart teams can immediately enhance their risk stratification capabilities by implementing systematic body composition analysis during routine pre-procedural CT review. Traditionally, body mass index (BMI) has been used as a simple measure of obesity in cardiovascular risk assessment. However, BMI alone fails to capture the nuances of body composition and fat distribution, which are increasingly recognized as important determinants of procedural outcomes [2,3]. This limitation is particularly relevant in the context of the “obesity paradox” observed in this population, where moderate obesity appears to confer a survival advantage [[4], [5], [6]]. Our recent systematic review of 14 studies demonstrates this relationship is far more nuanced than BMI can capture [1].

When structural heart teams examine adipose tissue characteristics during pre-procedural planning (Table 1), we found that higher subcutaneous adipose tissue (SAT) area/volume consistently associated with better survival across five studies (HR range: 0.83–2.77, *p* < 0.05) [3,4]. This enables teams to identify patients with potentially better procedural tolerance. More intriguingly, adipose tissue quality, measured by CT attenuation, proved equally important, with lower SAT and visceral adipose tissue (VAT) density correlating with improved outcomes (HR range: 1.31–1.46, *p* < 0.05) [3,5]. Teams can use these density measurements to stratify patients requiring enhanced peri-procedural monitoring protocols.

**Table 1:** CT-derived adipose tissue parameters and their prognostic value in TAVI patients.

Adipose parameter	Key findings	Effect size	Clinical implications
SAT area/volume	Consistently associated with better survival across multiple studies	HR range: 0.76–0.87 (higher SAT = lower risk) HR range: 1.99–2.77 (lower SAT = higher risk)	Lowest quartile patients may benefit from nutritional optimization; patients with moderate SAT show best outcomes
SAT density	Lower density consistently associated with better survival	HR range: 1.01–1.46 (higher density = higher risk)	Lower density indicates less fibrosis/inflammation and healthier adipocyte function; potential novel biomarker
VAT area/volume	Mixed results; relationship depends on obesity status	In non-obese: HR 2.30 (low vs. high VAT) In obese: HR 2.5 (high vs. low VAT)	Optimal VAT range may differ based on overall body composition; U-shaped relationship with outcomes
VAT density	Lower density consistently associated with better survival	HR range: 1.31–1.57 (higher density = higher risk)	Lower density reflects better metabolic profile; strongly associated with improved survival
VAT:SAT ratio	Ratio < 1 associated with better cardiovascular outcomes	HR 3.06 (ratio >1 vs. <1)	Simple ratio could serve as easily calculated prognostic marker; reflects metabolically healthier fat distribution
Total adipose tissue	Higher volume associated with better survival	HR 0.82 (high vs. low volume)	May reflect protective energy reserves during physiological stress; potential marker for absence of cardiac cachexia

This table summarizes key findings extracted from our systematic review [1]. Data from 14 studies comprising 9692 patients were analyzed. Adipose parameters were extracted from pre-TAVI CT scans at various anatomical levels (primarily L3 vertebra). Hazard ratios (HR) >1 indicate increased risk; ratios <1 indicate decreased risk. SAT = Subcutaneous Adipose Tissue; VAT = Visceral Adipose Tissue. Tissue density is measured by CT attenuation in Hounsfield Units, with lower density (more negative HU values) reflecting larger adipocytes with potentially healthier metabolic profiles. These findings demonstrate that adipose tissue characteristics provide significant prognostic information beyond traditional BMI measurements, with tissue quality (density) and distribution (VAT:SAT ratio) offering particularly valuable insights for risk stratification.

However, the relationship between adiposity and TAVI outcomes is not simply linear but demonstrates a U-shaped pattern that clinicians must understand for optimal patient selection. While higher SAT area/volume is generally associated with better survival, this applies primarily to patients moving from the lowest quartile toward moderate levels, not to unlimited SAT increase. The U-shaped pattern emerges when considering both extremes: patients in the lowest SAT quartile (potentially indicating cardiac cachexia or advanced heart failure) and those with morbid obesity both show worse outcomes compared to patients with moderate adiposity. This explains why ‘higher SAT = better survival’ coexists with the obesity paradox where morbid obesity worsens outcomes. The optimal range appears to be moderate subcutaneous adiposity that provides metabolic reserves without crossing into pathological obesity territory. This guides teams in identifying patients who may benefit from delayed procedures with interim optimization versus those ready for immediate intervention.

Meanwhile, specific fat distribution patterns provide insights impossible to detect with BMI measurements—a VAT:SAT ratio < 1 was associated with better cardiovascular outcomes (HR: 3.06, 95 % CI 1.20–7.77) [6], highlighting the importance of where fat is stored, not merely how much exists.

Perhaps most clinically relevant is the obesity-dependent effect observed by Mancio et al., where higher VAT was associated with better survival in non-obese patients (HR: 2.30, 95 % CI 1.12–4.91), while lower VAT was beneficial in obese individuals (HR: 2.5, 95 % CI 1.10–5.84) [7]. This context-dependent relationship enables structural heart teams to apply patient-specific risk assessment reflecting fundamental differences in metabolic physiology. In non-obese patients, modest VAT accumulation represents normal physiological adaptation and metabolic reserve capacity, providing protective energy stores during the physiological stress of TAVI procedures with preserved metabolic flexibility. Conversely, in obese patients, higher VAT levels indicate advanced metabolic dysfunction characterized by adipocyte hypertrophy, increased inflammatory cytokine production, insulin resistance, and ectopic fat deposition. In this population, lower VAT represents relatively better metabolic health within the context of existing obesity. This reflects the concept of ‘metabolically healthy obesity’—where fat distribution patterns, rather than total adiposity, determine metabolic consequences.

The clinical implications are substantial for structural heart teams. Pre-TAVI planning already includes CT imaging for anatomical assessment; extracting body composition data requires minimal additional resources but yields significant prognostic information. By incorporating these measurements into pre-procedural evaluation, clinicians could enhance risk stratification, potentially informing decisions about procedural timing, peri-procedural management, and post-TAVI care (Fig. 1). We have developed a novel decision algorithm (Fig. 2) that provides teams with standardized, sex-specific thresholds for implementing these measurements in clinical practice [8].

**Fig. 1:**
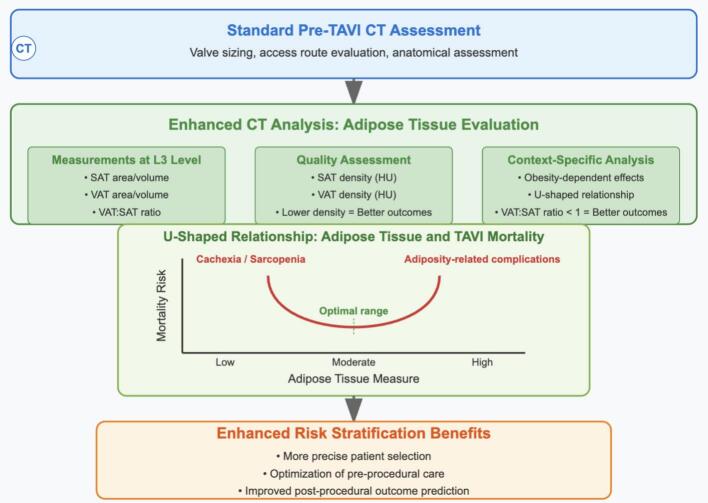
Adipose tissue analysis for TAVI risk stratification. This figure illustrates how standard pre-TAVI CT assessment can be enhanced with adipose tissue evaluation at the L3 level, including measurements of SAT/VAT area, tissue density, and distribution patterns. The U-shaped curve demonstrates that both extremely low and high adipose tissue measures correlate with worse outcomes, with an optimal middle range associated with better survival. Integration of these parameters into clinical workflow may enable more precise patient selection and personalized management without additional testing burden.

**Fig. 2:**
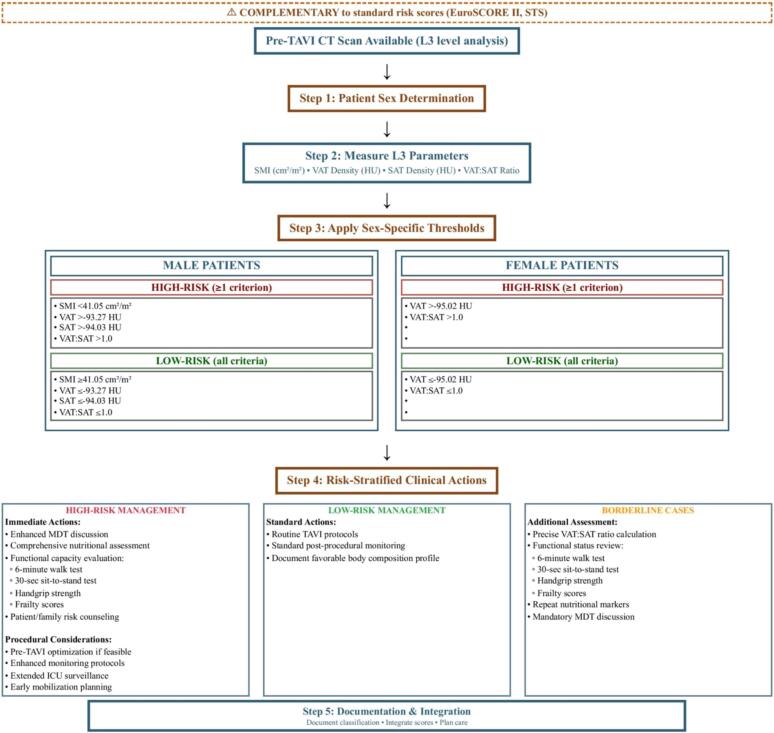
CT-derived body composition risk stratification algorithm for TAVI patients. The decision tree provides sex-specific thresholds for skeletal muscle index (S M I), visceral adipose tissue (VAT) density, subcutaneous adipose tissue (SAT) density, and VAT:SAT ratio measurements at the L3 vertebral level. Risk classification guides clinical decision-making and peri-procedural management strategies. Abbreviations: CT, computed tomography; HU, Hounsfield units; L3, third lumbar vertebra; MDT, multidisciplinary team; SAT, subcutaneous adipose tissue; SMI, skeletal muscle index; STS, Society of Thoracic Surgeons; TA VI, transcatheter aortic valve implantation; V AT, visceral adipose tissue. Data source: Risk thresholds adopted from [8]. Validation performed in Central European population; international validation recommended prior to widespread implementation.

For those considering these measurements in clinical practice, the decision algorithm offers immediate implementation guidance. L3 vertebral level assessment appears most promising based on our review, though protocols vary institutionally, and our decision tree provides specific thresholds validated in Central European population [8]. The algorithm stratifies candidates into high-risk and low-risk categories using skeletal muscle index, VAT density, SAT density, and VAT:SAT ratio measurements for men and VAT density and VAT:SAT ratio for women. High-risk candidates require enhanced multidisciplinary team discussion, comprehensive nutritional assessment, and extended surveillance protocols, while low-risk patients can proceed with standard care pathways. Software options range from commercial to emerging AI-based tools, with varying accessibility. While certain threshold values appeared meaningful in our analysis, these would benefit from validation across diverse populations before clinical application. The VAT:SAT ratio offers a relatively accessible starting point for centers with limited resources, though like all parameters, requires further validation. Any implementation would likely benefit from a gradual approach, beginning perhaps with research protocols and involving multidisciplinary expertise to determine feasibility within specific institutional contexts.

An important distinction must be made between the prognostic value of adipose tissue parameters and their potential as therapeutic targets. This commentary focuses specifically on risk stratification and patient selection rather than therapeutic intervention. The immediate clinical value lies in using these parameters as they exist at the time of TAVI evaluation. Analogously, we routinely use age, comorbidities, and frailty indices for risk stratification without expecting to modify them before intervention. The clinical utility of adipose tissue parameters operates independently of their modifiability—their value lies in identifying patients at different risk levels to inform procedural timing, peri-procedural management strategies, and patient counseling.

Current risk assessment tools for TAVI focus primarily on comorbidities, frailty indices, and procedural considerations. Incorporating body composition parameters could refine these models, potentially helping resolve clinical dilemmas in borderline candidates. For patients with low muscle mass but preserved subcutaneous adiposity, for instance, nutritional optimization might be prioritized over extended waiting periods that could exacerbate sarcopenia.

We acknowledge that SAT and VAT parameters may reflect general metabolic health rather than TAVI-specific pathophysiology. However, this does not diminish their clinical utility in TAVI risk stratification. The TAVI population represents a unique clinical context: elderly patients with severe comorbidities undergoing high-stress procedures where marginal physiological reserves become critically important. Many validated risk stratification tools (EuroSCORE, STS score) similarly incorporate general health markers rather than procedure-specific pathophysiology, yet remain clinically valuable. Most importantly, the clinical question is not whether these parameters reflect TAVI-specific mechanisms, but whether they enhance our ability to predict outcomes and optimize patient selection—which the evidence clearly supports.

Despite compelling evidence, implementation challenges exist. Measurement techniques vary across studies, with different anatomical reference points (L3 vertebra versus umbilical level) and software applications used [9,10]. Standardization is critical—our review suggests measurements at the L3 level show strongest correlation with whole-body composition and most consistent prognostic associations.

The practical question for clinicians becomes: if this prognostic information already exists in routine pre-TAVI scans, can we afford to ignore it? As Guler et al. demonstrated, higher total adipose tissue volume was significantly associated with better survival outcomes (HR: 0.82, 95 % CI 0.71–0.95) [11].

These complex, non-linear relationships underscore why traditional BMI measurements inadequately capture cardiovascular risk in TAVI populations. The optimal adipose tissue profile appears to be moderate subcutaneous adiposity with minimal visceral accumulation—providing adequate metabolic reserves without inflammatory complications. However, this optimal profile differs between patient populations: non-obese patients may benefit from modest VAT as metabolic reserve, while obese patients benefit from VAT minimization to reduce inflammatory burden.

Future research should focus on standardizing adipose tissue measurement protocols at the L3 vertebral level and establishing clear thresholds for clinical decision-making. While prospective interventional studies would strengthen the evidence base for modifying adipose tissue parameters to improve TAVI outcomes, this limitation does not negate the immediate clinical value of these parameters for risk stratification. Many established risk assessment tools in cardiovascular medicine (GRACE score, CHADS2-VASc, frailty indices) gained clinical adoption based on their prognostic accuracy rather than interventional validation. Artificial intelligence approaches, as demonstrated in our recent work [8], may further streamline analysis and integration into clinical workflows (Fig. 3).

**Fig. 3:**
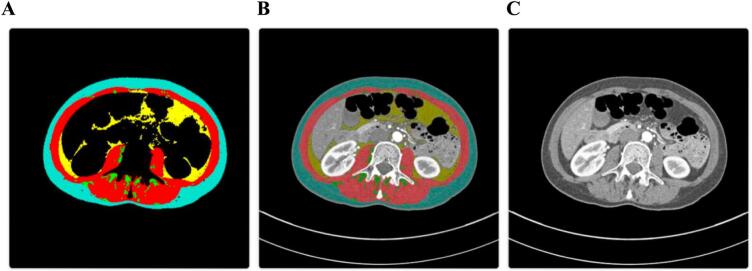
AI-automated tissue segmentation at the third lumbar vertebra (L3) demonstrating sarcopenic phenotype in a 51-year-old female patient (BMI 18.94). (A) Segmentation results using AI-based algorithm showing tissue classification: Skeletal muscle (red, reduced area indicative of sarcopenia); Visceral adipose tissue (yellow, VAT_CSA: 31.24 cm^2^); Subcutaneous adipose tissue (turquoise, SAT_CSA: 73.79 cm^2^, reduced layer); Intramuscular adipose tissue (not visible, IMAT_CSA: 7.24 cm^2^); Excluded regions (black). (B) Overlay of segmentation map on the original CT image demonstrating anatomical correspondence of tissue classification. (C) Original axial CT image at L3 level. This automated approach quantifies body composition parameters at L3 level, which our research identifies as having significant prognostic value in TAVI patients. The segmentation methodology employs neural networks for automated analysis of clinically acquired CT scans [11], enabling the extraction of adipose tissue characteristics with demonstrated prognostic value. The patient demonstrates a skeletal muscle index (SMI) of 34.07 cm^2^/m^2^, confirming sarcopenia in female patients. On the other hand, the adipose tissue quality, measured by CT attenuation (SAT_HU: −100.07, VAT_HU: −94.66), reflects lower density values that our systematic review has linked with improved survival. These measurements extracted from routine pre-procedural CT scans offer valuable prognostic information without additional radiation exposure, potentially enhancing risk stratification for patients undergoing TAVI procedures. Abbreviations: VAT_CSA: Visceral Adipose Tissue Cross-Sectional Area; SAT_CSA: Subcutaneous Adipose Tissue Cross-Sectional Area; IMAT_CSA: Intramuscular Adipose Tissue Cross-Sectional Area; L3: Third Lumbar Vertebra; TAVI: Transcatheter Aortic Valve Implantation; SMI: Skeletal Muscle Index; HU: Hounsfield Units (CT attenuation measure); BMI: Body Mass Index; CT: Computed Tomography. (For interpretation of the references to colour in this figure legend, the reader is referred to the web version of this article.)

The transition from research evidence to clinical practice represents a critical gap in modern cardiology. This commentary specifically addresses the implementation challenge: how can TAVI centers immediately enhance their risk stratification using data already present in their routine imaging protocols? The answer lies not in conducting additional studies, but in optimizing the utilization of existing clinical data. Every pre-TAVI CT scan contains valuable adipose tissue information that could inform patient selection, procedural planning, and post-procedural care strategies.

As TAVI expands across a wider spectrum of patients, from the frailest elderly to those with moderate surgical risk, optimizing patient selection becomes increasingly important. The data is already there in your CT scans. The only question that remains is: are you looking?

## CRediT authorship contribution statement

**Marek Kantor:** Writing – original draft, Investigation, Data curation. **Otakar Jiravsky:** Writing – review & editing, Validation, Methodology. **Matej Pekar:** Writing – review & editing, Visualization, Supervision, Conceptualization.

## Ethical approval

Ethical approval was not required for this commentary article as it primarily provides commentary on previously published research, specifically our systematic review that analyzed published literature on CT-derived adipose tissue parameters in TAVI patients. The CT segmentation image presented in Fig. 3 was obtained and processed in compliance with our ethics committee agreement approved by the Hospital AGEL Trinec-Podlesi Ethics Committee (EK 301/22).

## Declaration of Generative AI and AI-assisted technologies in the writing process

During the preparation of this work the authors used Claude Sonnet 4 (Anthropic) in order to assist with the writing process. After using this tool, the authors reviewed and edited the content as needed and take full responsibility for the content of the publication.

## Funding information

This study was supported by Specific University Research Grants no. MUNI/A/1641/2024 provided by the Ministry of Education, Youth, and Sports of the Czech Republic, and Internal grant of AGEL no. INT2023001.

## Declaration of competing interest

The authors declare that they have no known competing financial interests or personal relationships that could have appeared to influence the work reported in this paper.
